# The complete mitochondrial genome of loliginid squid (*Uroteuthis chinensis*) from Minnan–Taiwan bank fishing ground

**DOI:** 10.1080/23802359.2019.1703599

**Published:** 2020-01-08

**Authors:** Lei Xu, Xuehui Wang, Feiyan Du

**Affiliations:** a Fishery Environment Research Laboratory, South China Sea Fisheries Research Institute, Chinese Academy of Fishery Sciences, Guangzhou, China;; b Guangdong Provincial Key Laboratory of Fishery Ecology and Environment, Chinese Academy of Fishery Sciences, Guangzhou, China

**Keywords:** Mitochondrial genome, *Uroteuthis chinensis*, Minnan–Taiwan bank fishing ground

## Abstract

The squid *Uroteuthis chinensis* is commercially important fishery species in many coastal regions of Asia. In this study, we described the complete mitochondrial genome of *U. chinensis*. The genome is 17,353 bp in length, encoding the standard set of 13 protein-coding genes, 22 transfer RNA genes, two ribosomal RNA genes, with circular organization. The overall base composition of the whole mitochondrial genome was A (39.56%), T (31.71%), G (9.05%) and C (19.68%) with an AT bias of 71.27%. The longest protein-coding genes of these species was *ND5*, whereas the shortest *ATP8*.

*Uroteuthis chinensis* (Gray, 1849) is a large-sized Indo-Pacific species extending from the western Pacific to Indian Ocean (Natsukari and Tashiro 1991). It was formerly classified under the genus *Loligo* (Lamarck, 1798), as *Loligo chinensis*. *Uroteuthis chinensis* support a very important commercial fisheries industry in China on the continental shelf off Guangdong, southern Fujian and around the Pescadores Islands in the Taiwan Strait (Voss and Williamson 1971). *Uroteuthis chinensis* accounts for up to 90% of the loliginid catch in several parts of China and 15–40% of the trawl catch in the Gulf of Thailand. Here, we sequenced and annotate mitogenome of *U. chinensis* form Minnan–Taiwan bank fishing ground (South China Sea) to provide molecular information for genetically understanding of loliginid squid.

The specimens of *U. chinensis* were collected from Minnan–Taiwan bank fishing ground (South China Sea) (22°30′N, 118°45′E) in 19th September, 2018. Whole genomic DNA was extracted from muscle tissue of one specimen of *U. chinensis* using TIANamp Marine Animals DNA Kit (TIANGEN, China). The concentration for use as a PCR template was adjusted to an A_260_ of about 0.05 to 0.2. The collected specimen and extracted DNA were stored in Guangdong Provincial Key Laboratory of Fishery Ecology and Environment (specimen accession number: MT2018-A-22). The complete mitochondrial genomes of *U. chinensis* was sequenced using PCR primers designed from highly conserved regions of transfer RNA (tRNA) sequences of related species (Jiang et al. 2016) with additional specific primers designed as required from sequences already obtained. Long-PCR amplifications were performed by thermo-cycling using five pairs of primers and PCR amplicons were subjected to build up genomic library and pair-end sequencing by MiSeq. The COI sequence of *U. chinensis* was used as reference seeds for iterative assembly by MITObim v.1.8 (Hahn et al. 2013). SeqMan v.7.1.0 was used for the mitogenome assembly and annotation (Swindell and Plasterer 1997). Transfer RNA genes were predicted using online software tRNAScan-SE 1.21 (Lowe and Eddy 1997). All protein-coding gene (PCGs) are aligned independently, then concatenated to be applied for phylogenetic reconstruction with other cephalopods in MrBayes v 3.12 (Ronquist and Huelsenbeck 2003) using relaxed clock model.

The *U. chinensis* mitochondrial genome forms a 17,353 bp closed loop (GenBank accession number MN687903). The overall base composition of the whole mitochondrial genome was A (39.56%), T (31.71%), G (9.05%) and C (19.68%) with an AT bias of 71.27%. This mitochondrial genome represents a typical *Uroteuthis* mitochondrial genome and matches with the *U. duvaucelii* genome (Jiang et al. 2016), in which it comprises 13 protein-coding genes, 22 transfer RNA genes and 2 ribosomal RNA genes (12S rRNA and 16S rRNA). The ATG initiation codon are used in all protein-coding genes except *ND2*, *ND6* and *ND5* (ATT), and the stop codons of all the 13 protein-coding genes were complete. Ten protein-coding genes (*ND3*, *CYTB*, *ND6*, *ND1*, *ND2*, *COX1*, *COX2*, *ATP8*, *ATP6,* and *COX3*) use TAA as the termination codon; three protein-coding genes (*ND4*, *ND5*, *ND4L*) use TAG as the termination codon. Meanwhile, the longest protein-coding genes of these species was *ND5* (1644 bp), whereas the shortest *ATP8* (153 bp). *lrRNA* and *srRNA* genes are 1398 bp and 929 bp in length separately. All the 22 typical tRNAs possess a complete clover leaf secondary structure, ranging from 65 bp to 79 bp. The Bayesian inference phylogenetic tree showed that *U. chinensis* firstly grouped with species of *U. edulls* (Figure 1). We have the confidence to construct phylogenetic trees, based on the complete the mitochondrial genomes, but the evolution history of marine cephalopod still needs future research to be clearly resolved.

**Figure 1.**
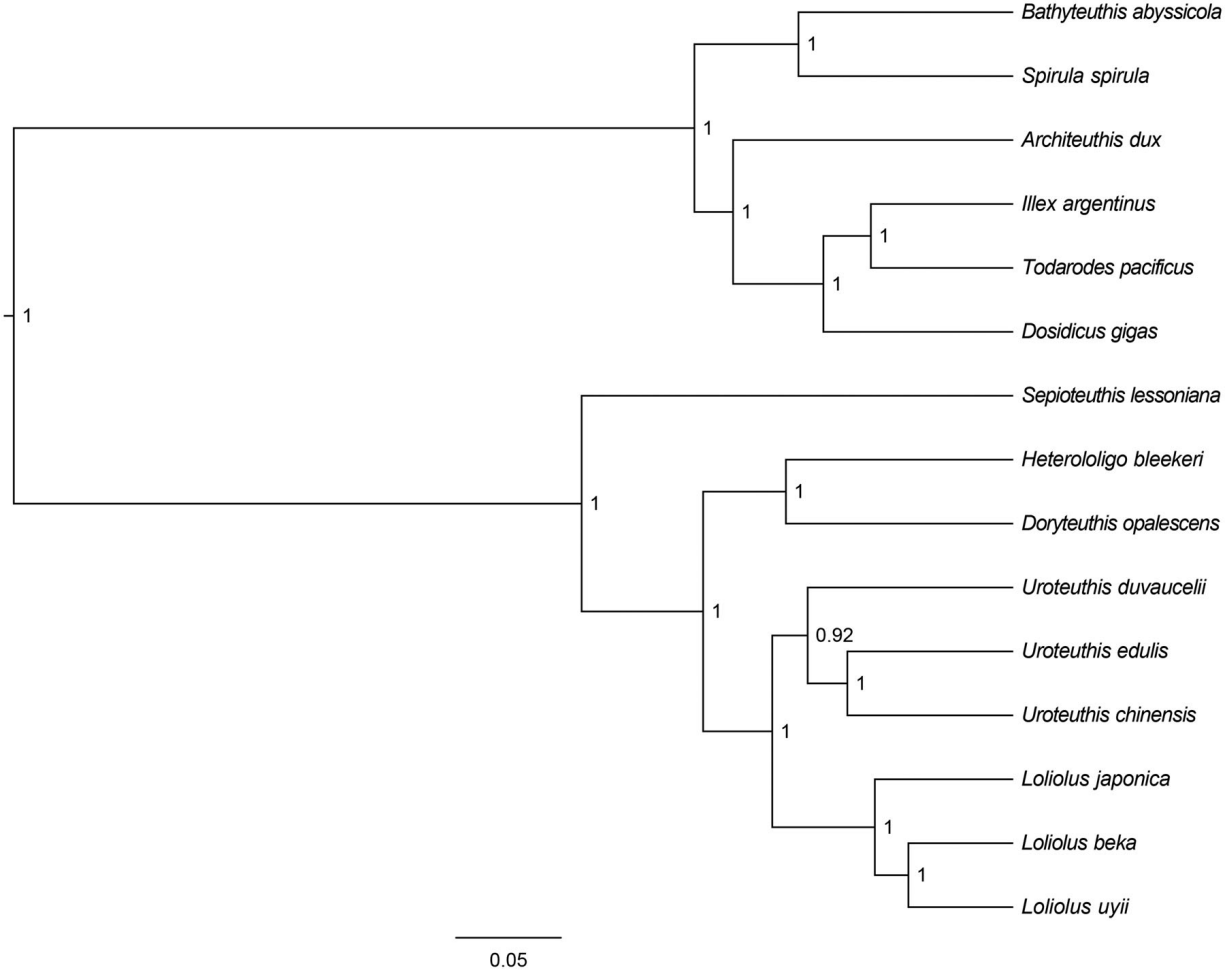
The Bayesian inference phylogenetic tree for Cephalopoda based on mitochondrial PCGs and rRNAs concatenated dataset. The gene’s accession numbers for tree construction are listed as follows: *Loliolus japonica* (KU568467), *Heterololigo bleekeri* (AB029616), *Uroteuthis eduli*s (AB675081), *Loliolus uyii* (KP265013), *Uroteuthis duvaucelii* (KR051264), *Doryteuthis opalescens* (KP336703), *Loliolus beka* (KT254309), *Sepioteuthis lessoniana* (KM878671), *Illex argentines* (KP336702), *Dosidicus gigas* (EU068697), *Spirula spirula* (KU893141), *Bathyteuthis abyssicola* (AP012225), *Todarodes pacificus* (AB158364), *Architeuthis dux* (KC701764).

## References

[CIT0001] Hahn C, Bachmann L, Chevreux B. 2013. Reconstructing mitochondrial genomes directly from genomic next-generation sequencing reads-a baiting and iterative mapping approach. Nucleic Acids Res. 41(13):e129. 23661685 10.1093/nar/gkt371 PMC3711436

[CIT0002] Jiang L, Ge C, Liu W, Wu C, Zhu A. 2016. Complete mitochondrial genome of the *Loligo duvaucelii*. Mitochondrial DNA A. 27(4):2723–2724. 10.3109/19401736.2015.1046164 26104158

[CIT0003] Lowe TM, Eddy SR. 1997. tRNAscan-SE: a program for improved detection of transfer RNA genes in genomic sequence. Nucleic Acids Res. 25(5):955–964. 9023104 10.1093/nar/25.5.955 PMC146525

[CIT0004] Natsukari Y, Tashiro M. 1991. Neritic squid resources and cuttlefish resources in Japan. Mar Behav Physiol. 18(3):149–226.

[CIT0005] Ronquist F, Huelsenbeck JP. 2003. MrBayes 3: Bayesian phylogenetic inference under mixed models. Bioinformatics. 19(12):1572–1574. 12912839 10.1093/bioinformatics/btg180

[CIT0006] Swindell SR, Plasterer TN. 1997. Seqman, contig assembly. In: Swindell SR, editor. Sequence data analysis guidebook. Totowa (NJ): Springer; p. 75–89. 9089604

[CIT0007] Voss GL, Williamson GR. 1971. Cephalopods of Hong Kong. Hong Kong (China): Hong Kong Government Press.

